# Caloric Vestibular Stimulation Reduces Pain and Somatoparaphrenia in a Severe Chronic Central Post-Stroke Pain Patient: A Case Study

**DOI:** 10.1371/journal.pone.0151213

**Published:** 2016-03-30

**Authors:** Grazia Fernanda Spitoni, Giorgio Pireddu, Gaspare Galati, Valentina Sulpizio, Stefano Paolucci, Luigi Pizzamiglio

**Affiliations:** 1 Department of Psychology–Sapienza University of Rome, Rome, Italy; 2 Laboratory of Neuropsychology, IRCCS Santa Lucia Foundation, Rome, Italy; French National Centre for Scientific Research, FRANCE

## Abstract

Central post-stroke pain is a neuropathic syndrome characterized by intolerable contralesional pain and, in rare cases, somatic delusions. To date, there is limited evidence for the effective treatments of this disease. Here we used caloric vestibular stimulation to reduce pain and somatoparaphrenia in a 57-year-old woman suffering from central post-stroke pain. Resting-state functional magnetic resonance imaging was used to assess the neurological effects of this treatment. Following vestibular stimulation we observed impressive improvements in motor skills, pain, and somatic delusions. In the functional connectivity study before the vestibular stimulation, we observed differences in the patient’s left thalamus functional connectivity, with respect to the thalamus connectivity of a control group (N = 20), in the bilateral cingulate cortex and left insula. After the caloric stimulation, the left thalamus functional connectivity with these regions, which are known to be involved in the cortical response to pain, disappeared as in the control group. The beneficial use of vestibular stimulation in the reduction of pain and somatic delusion in a CPSP patient is now documented by behavioral and imaging data. This evidence can be applied to theoretical models of pain and body delusions.

## Introduction

Central post-stroke pain (CPSP), previously known as thalamic pain syndrome of Déjerine and Roussy, is a condition developed after a thalamic stroke.

It has been considered an expression of central pain, which is mostly defined as central neuropathic pain generated by alterations of the central nervous system. As reviewed by Henry et al. (2008), Déjerine and Roussy described the CPSP “among the most spectacular, distressing, and intractable of pain syndromes” [1]. In fact, even though the pain is commonly reported as acute and intolerable, other sensory symptoms are vague and frequently hard to describe. Indeed, patients may show dysesthesia (painful and persistent sensation induced by a gentle touch of the skin), allodynia (pain due to stimulus that does not normally elicit any) and hyperalgesia (excessive sensitivity and a raised threshold to painful stimuli).

In rare cases, CPSP has also been associated with symptoms of somatic delusions that mirror the somatoparaphrenic syndrome. Briefly, somatoparaphrenia is a delusional belief whereby a patient feels that a damaged limb does not belong to his body or it presents salient deformities [2–3]. Hanihara and colleagues (2009) described a rare case of an unusual association of specific somatosensory delusions (little running worms infesting the mouth) and CPSP after left-posterior thalamic hemorrhage [4]. The authors suggested that the presence of this kind of somatic delusions was caused by the disruption of the somatosensory pathway and that the subsequent cortical sensory deafferentation and reorganization arising from this disruption, contributed to the development of delusional parasitosis.

Unfortunately, there is little evidence that pharmacological or neurostimulation treatments effectively reduce CPSP symptoms [5–6]. A convincing option proposed in the last 15 years, is the use of Caloric Vestibular Stimulation (CVS) as a method to reduce neuropathic symptoms. For example Bottini et al. (2005), found that the CVS administered to the left ear produced a transient remission of right hemianesthesia in a left brain damage patient [7]. The, beneficial use of cold CVS was also reported by Rode et al. [8] who found that, in a patient affected by left lateral medullary stroke with left facial macrosomatognosia and neuropathic pain, left CVS abolished the facial somatosensory illusion but it had no effect on the left facial pain. Finally and of particular interest for this study convincing findings have been reported by Ramachandran et al. (2007) and McGeoch and colleagues (2008) who used Caloric Vestibular Stimulation (CVS) to treat contralesional body pain caused by CPSP [9–10]. These authors found that in some patients the pain decreased after CVS, whereas placebo had no effect. This evidence has been discussed in light of the thermosensory disinhibition hypothesis [11–12].

The basis of the thermosensory disinhibition hypothesis is that central pain is a thermoregulatory disorder that occurs from the loss of the central inhibition of pain by cooling. As reported by McGeoch and colleagues, temperatures below 25uC activate both the cold thermoreceptors (Aδ fibre) and the nociceptors (C-fibre) [10]. Then these inputs pass to the thalamus via the lamina I layer: the Aδ fibre input goes through the posterior part of the ventromedial nucleus (VMpo) in the lateral thalamus to the dorsal posterior insula (dpIns), whereas the C fibre input passes via the ventral caudal part of the medial dorsal nucleus of the thalamus to the anterior cingulate cortex (ACC). Usually, the Aδ fibres activate the dpIns to suppress the perception of pain at the ACC but when the temperature decreases under 15uC, the C activity predominates over the Aδ activity. Consequently, the ACC is no longer suppressed and hypothetically cold temperatures are perceived as pain. Therefore it is realistic that lesions of the lamina I spino-thalamo-cortical pathway terminating in dpIns, can disinhibit a thermosensory network comprising the parabrachial nucleus (PB), the periaqueductal grey (PAG) and the ACC.

Turning back to the thermosensory disinhibition hypothesis, Craig (1998), proposed that lesions of the lamina I pathway can cause an imbalance in the bilateral integration of thermosensation in the brainstem by the PB and PAG and the development of CPSP [11–12]. Given this theoretical framework, it seems that CPSP occurs due to an imbalance, at the level of the brainstem, in the integration of thermosensory information from each hemisphere. McGeoch and colleagues argued that the CVS could rebalance this integration, by the temporary activation of the parieto-insular vestibular cortex [10].

Here we have described a rare case of chronic CPSP syndrome associated with severe somatoparaphrenic delusions, in a 57 year old woman. In an attempt to reduce pain and somatoparaphrenia, we treated the patient with CVS and assessed the effect of this treatment over motor skills, pain and somatic delusions. We finally evaluated whether CVS-induced changes in the intrinsic functional architecture of the patient’s brain, using a resting-state functional connectivity study involving a control group.

## Methods

### Case report

SF is a 57–year–old woman with CPSP–associated chronic somatoparaphrenia. At the age of 52, SF had a left hemispheric stroke, which provoked hemiplegia on the right side of her body. However, her motor impairment approached complete remission after 14 months of therapy. At age of 54, she experienced a second left hemispheric stroke. In the second episode, an ischemic stroke damaged the left thalamus causing an initial lack of sensation and tingling in the right side of the body together with sever motor impairments of the right limbs. Indeed, ten months after the second stroke, the numbness developed into severe and chronic pain. We have met SF three years after the second stroke. At that time SF suffered from intense pain, motor impairment of her right side, and severe somatoparaphrenia. The pain was intolerable and refractory to standard chemical medications. SF reported a transitory pain relief only when treated with opiates; unfortunately the common side effects of opiates prevented the everyday use. The motor impairment involved the right limbs; the movement fluidity of the arm and of the leg was compromised and SF could walk slowly with the help of a crutch. Concerning the somatoparaphrenic symptoms, she reported the following delusions: her right arm was attached to her hip, her right leg had elongated to her neck, her teeth could run inside her mouth and sometimes they were rearranged and reached her right foot, the right side of her face was expanded and heavy. In order to check the size of her face and the presence of the teeth in the mouth, she used to look herself into a big mirror that she kept hidden in her bag. She also reported her symptoms in the following way: “Sometimes I feel my right arm strange, maybe it is not mine anymore.” The patient also suffered from mild dysarthria linked to facial paresis.

A neuropsychological examination indicated that the patient did not suffer from any language or spatial impairment. Indeed, the patient’s intelligence was within a normal range. Also tactile perception, thermal sensitivity of limbs and face were preserved. (Von fray-2PD-thermal sensitivity) (Table 1 summarizes the neuropsychological assessment; references details in S1 Ref).

**Table 1:** Neuropsychological test scores and performance on tactile acuity tests of S.F.

	Tests	S.F.’s Score (ES)	Norms and controls*
Orientation	Space	normal	
	Time	normal	
Attention	Visual Search	0	>3
	Go-no go test	4	>3
Language	Token Test	4	>3
	Fluency (semantic)	3	>3
	Fluency (lexical)	3	>3
Apraxia	Ideomotor	3	>3
	Ideational	4	>3
	Constructional	4	>3
Agnosia	Categorical	4	>3
	Functional	4	>3
Memory	Corsi Span	2	>3
	Verbal Span	1	>3
Executive Functions	BADS	4	> 1
	TOL	28	> 9
WAIS-R	VIQ	107	>75
	PIQ	96	>75
	TIQ	103	>75
Tactile acuity tests	2Point Discrimination	3.21	3.54 (1.09)*
	Von Frey	2.69	2.77 (0.27)*
	Thermic (in °C)	36.52	36.9 (7.17)*

**ES** = Equivalent Score = **0 = Severe impairment; 1 = Impairment; 2 = Moderate impairment; 3 = Limits of normal; 4 = Normal**BADS = Behavioural AssessmentSssessment of Dysexecutive Syndrome, **TOL** = Tower of London. **WAIS-R** = Wechsler Adult Intelligence Scale revised.

*Control Group Performance = 26 healthy volunteers (mean age 38, SD ±1.15; 15 female)

**Fig 1:**
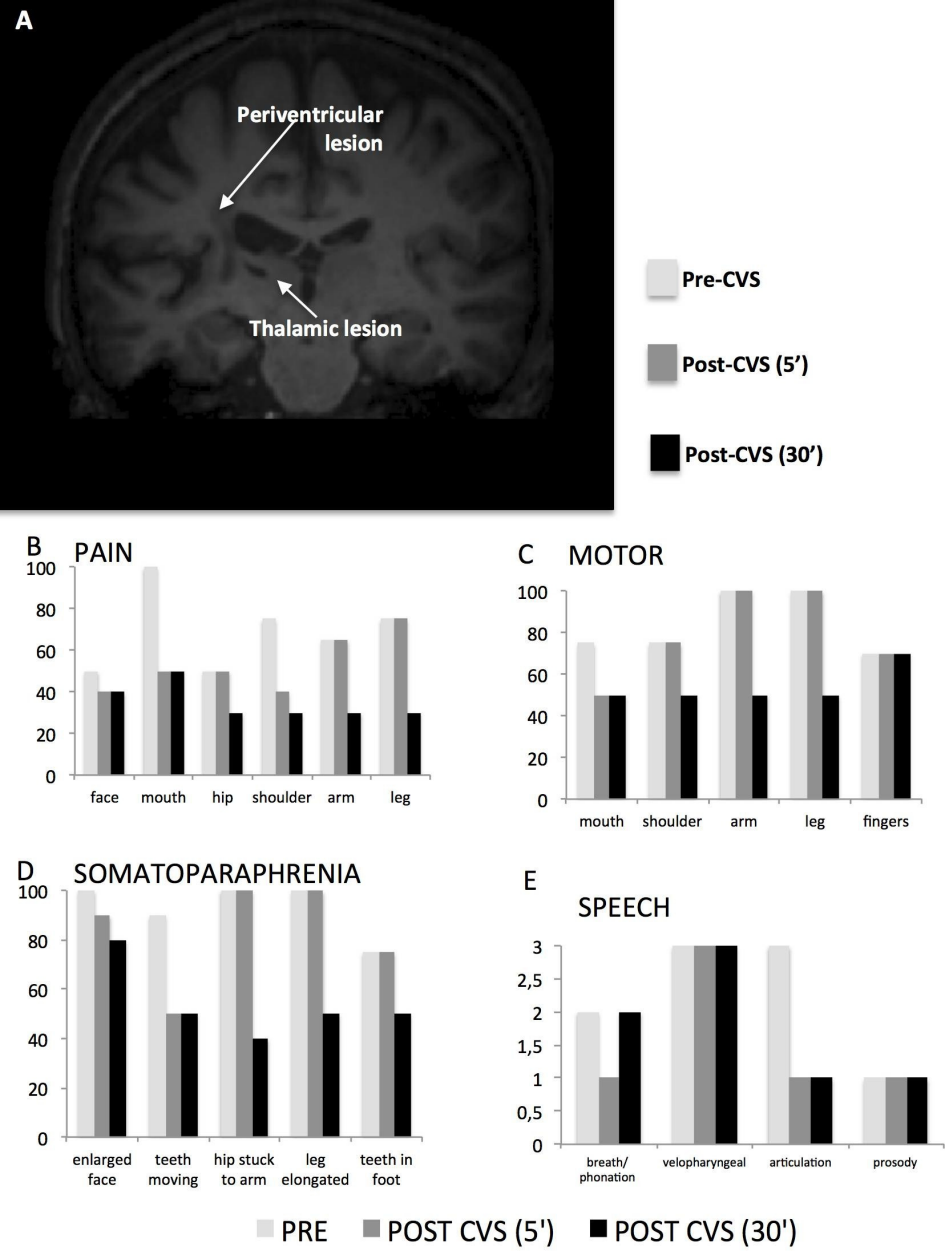
MRI scan of patient SF and behavioral results before and after CVS. (A) T1–weighted sequence reveals subcortical lesions that were also present in a small portion of the left lateral thalamus. (B–E) The effects of CVS were determined for several symptoms including pain (B), motor function (C), somatoparaphrenia (D), and articulation (E). All the histograms refer to the patient’s subjective evaluation. In graphs B-C-D, the Y axis shows the level of impairment (0 = no impairment; 100 = totally impaired). In graph E Y axis = 0 excellent (no altered articulation characteristics), 1 slight disorder, 2 moderate disorder, 3 severe disorder]. In all panels, post–CVS (5’) refers to measurements collected immediately after CVS, and post–CVS (30’) refers to measurements collected 30 minutes after the CVS.

An MRI scan revealed subcortical lesions in the white matter at the level of the corona radiata and the centrum semiovale of the left hemisphere. The periventricular lesion included a very small portion (volume = 68 mm3) of the left lateral thalamus (see Fig 1A); see also S1 Fig in Supporting Information).

At the time of the study SF was under pregabalin medication (Lyrica). This treatment was selected as a first choice medication suggested by European Guidelines for Neuropathic Pain.

It is important to say that even though pregabalin medication may produce some side effects (i.e. drowsiness, peripheral edema, fatigue, constipation, weight gain, blurred vision, ataxia, dizziness, headache, diplopia, xerostomia,.....) the Lyrica do not involve delusions.

#### Preliminary study

As described above SF suffered from an unbearable pain that in some occasions prevented her even from basic movements. For this reason, in order to evaluate the degree of feasibility that the patient could have gone through during the CVS experimental sessions, we implemented a preliminary study in which we evaluated the practicability of the protocol.

To this aim, we tested the patient as follows: SF was asked to walk to a folding bed and she was helped to lay down; the patient’s head was tilted about 30° forward using a common cushion; she was asked whether she felt confortable in that position; patient’s right ear was irrigated with about 30cc of body temperature water; in the supine position the patient was asked to describe her physical sensations; patient was helped to raise from the bed and again she was asked to describe and to evaluate her physical conditions; patient was helped to get out of bed and she was asked if she could walk to the chair; while sitting on the chair the patient was asked to refer any modification of the symptoms and/or any discomfort due to the technique.

The same procedure was carried out after two days but this time we irrigated the left ear. As expected the procedure was well tolerated by the patient. For what concerns the effect of the stimulation, we did not observe either the vegetative sensations or the nystagmus, confirming previous evidence on the use of body temperature water [13]. In respect to the physical sensations after the stimulations, the patient did not refer any amelioration of the symptoms. After this trial we asked SF whether she felt confortable with the procedure and she accepted the experimental protocol.

#### Experimental protocol

SF was evaluated in one-month time. Each session consisted in an experimental trial of about 3.5 hours followed by a monitoring of three more hours (see Table 2). In all these phases of the assessment, we asked SF to go through motor and verbal tasks and to score the intensity of pain and somatosensory delusions.

**Table 2:** Timeline of the experimental protocol

Session	Pre Assessment	fMRI Pre	CVS	fMRI Post	Post Assessment			Monitoring	
				5 minutes > 1 hour	5 min	30 min.	60 min	3 hours	5 hours
1	**+**		right ear		**+**	**+**	**+**	**+**	**+**
2	**+**		left ear		**+**	**+**	**+**	**+**	**+**
3	**+**		right ear		**+**	**+**	**+**		
4	**+**		left ear		**+**	**+**	**+**	**+**	**+**
5	**+**	**+**	left ear	+	**+**	**+**	**+**		

In the mouth motor task’, SF was required to: open and close the mouth; make a smile and simulate a kiss smacking. In the shoulder motor task, we asked SF to rotate the shoulder and to raise and lower it. Concerning the fingers, SF was required to tap each finger with the thumb; to snap and to clap. In the motor task with the arm, the movements required were: raise and lower one arm; raise and lower both arms together; touch the cheek with one hand; touch the cheeks with both hands; put the hand on the head. Finally, in the leg motor task, we asked to lift up the leg; to bend and raise the knee and to walk with and without the crutch. Every movement was repeated three times and the patient was asked to score her performance soon after the third repetition.

The intensities of pain and somatosensory delusions were evaluated with a series of Visual Analogic Scales (VASs). We used a horizontal line, 100 mm in length, anchored by word descriptors at each end (Pain = no pain / very severe pain; Somatoparaphrenia = no delusion / very strong delusion). The VASs employed in the evaluation of the Motor task were delimitated by the words easy movement / difficult movement.

SF was required to mark the point on the line that she felt represented her perception of her current state. The VAS score was then determined by measuring in millimetres from the left hand end of the line to the point that the patient marked.

The verbal abilities were assessed with the Assessment of Motor Speech for Dysartria–AMSD [14]. The monitoring phases consisted in a structured interview about motor and verbal abilities, intensity of pain and somatosensory delusions (the entire structured interview is reported in S1 Interview).

Before starting the experimental sessions we asked SF weather she felt able to go through the assessment. In three meetings out of eight, she refused to participate due to the intolerable pain. SF and control participants provided written informed consent for the study and SF signed the permission to videotape all the experimental sessions. The procedures performed in the study and the informed consent have been approved by Research Ethics Committee of the IRCCS Santa Lucia Foundation of Rome (CE-PROG.463) in accordance with the 1964 Helsinki declaration and its later amendments or comparable ethical standards.

#### Caloric vestibular stimulation study

As showed in Table 2, SF was first assessed with the experimental protocol described above and then she received the Caloric Vestibular Stimulation (CVS). After the CVS she received three more complete assessments and two monitoring.

The interval between the CVS sessions was about 4/5 days. We started with the irrigation of the right ear and after 4 days we tested the left ear. This procedure was completed two times in 20 days.

**Caloric Vestibular Stimulation:** We used ice water to stimulate the patient’s ears. Specifically, we irrigated the external ears canal with 30 cc of cold water (4°C) for 60 seconds. During stimulation, room lighting was neutral and the patient laid down on a folding bed with her head tilted approximately 30° forward.

In healthy subjects cold CVS in the ear generates a horizontal nystagmus with a leftward or rightward slow phase lasting about 3 min, and a marked sensation of vertigo. In our patient only the vestibular stimulation of the left ear immediately produced a transient horizontal nystagmus with a leftward slow phase. Moreover, SF experienced self-motion and vertigo without vegetative sensation or nausea for about 3 minutes.

The stimulation of the right ear did not show nystagmus and the vegetative sensations were mild.

**Scoring of the CVS effects:** In order to have more objective measures of motor functions and speech, we asked a group of 39 participants, blind to the study conditions, to score the patient’s performances. Each subject was asked to watch two videotapes (video PRE-CVS; video POST-CVS) and to score motor fluidity and verbal variables. Observers were split into two subgroups: one group first received the PRE-CVS video and then the POST-CVS video; the other group received the POST-CVS video before the PRE-CVS video. Data were then collected and analyzed by an independent experimenter blind to the study conditions.

#### fMRI study

The patient underwent two scanning sessions (pre and post CVS) in the same day, and the imaging procedure was identical across both sessions. SF was first assessed with the experimental protocol and then she went into the scanner for the first fMRI session. Then she received the CVS in the left ear and after 5 minutes she was scanned for the second fMRI session. At the end of the fMRI protocol the patient received two more assessments.

In order to explore the pattern of cortical connections associated with the thalamus, two fMRI resting-state scans were ran. This was crucial to characterize the resting-state functional connectivity of the perilesional thalamic area with the rest of the patient’s brain and compare it with an healthy control group (20 subjects; ten females) across the two sessions of CVS.

**Imaging parameters:** A Siemens Allegra scanner (Siemens Medical Systems, Erlangen, Germany), operating at 3T and equipped for echo planar imaging, was used to acquire functional magnetic resonance images. Head movements were minimized by mild restraint and cushioning. Functional MRI images of the entire cortex were acquired using BOLD imaging (30 slices, in–plane resolution = 3 x 3 mm, slice spacing = 4·5 mm, repetition time [TR] = 2 s, echo time [TE] = 30 ms, flip angle = 70 deg). We also acquired a three–dimensional, high–resolution, T1–weighted structural image (Siemens MPRAGE, 176 slices, in–plane resolution = 0·5 x 0·5 mm, slice thickness = 1 mm, TR = 2 s, TE = 4·38 ms, flip angle = 8 deg) and axial fluid-attenuated inversion recovery (FLAIR) sequences (TR = 8500, TE = 109, inversion time = 200) covering the whole brain.

We acquired resting-state fMRI scans of 128 functional MR images, lasting approximately 9 min, to explore the patient’s functional connectivity and to compare it with that of a healthy control group. During the resting state scans, participants were instructed to close their eyes and remain passive.

**Images preprocessing and analysis:** Image analysis was performed using SPM8 (http://www.fil.ion.ucl.ac.uk/spm). The first four volumes of each run were discarded to allow for T1 equilibration. All images were corrected for head movement (realignment) using the first volume as reference. Furthermore, the ArtRepair toolbox (version 4- http://spnl.stanford.edu/tools/ArtRepair) was used to correct for movement artifacts. The images of the patient were then co–registered onto her T1 image.

**Resting-state functional connectivity:** Images were preprocessed as described above. Further, co–registered images were normalized to the standard MNI–152 EPI template using the mean realigned image as a source to allow the comparison between SF and healthy participants.

Each preprocessed resting image was band–pass filtered to retain signals between 0.01 and 0.1 Hz, thus removing artifacts of linear drift and high–frequency noise. The thalamus time course was extracted from an anatomically–defined reconstruction based on the automatic segmentation of the patient’s brain. This region (regional peak: x = –25, y = –31, z = –6; size = 294 voxels; lesional voxels were excluded) was used as a seed to assess its “intrinsic” functional connectivity. This “seed–based” approach can be applied with a general linear model (GLM)—i.e. using time course regressors derived from the selected brain region to find voxels having correlated BOLD signal activity patterns. Time courses from the white matter and cerebral spinal fluid were used as confounding explanatory variables. For SF, a voxel–wise map, reflecting the functional connectivity between the left thalamus time course and the time course of all other brain voxels was created for each experimental session (pre–and post–CVS).

For the control group, a voxel-wise connectivity map was created for each participant, regardless of the experimental session, since they did not undergo any CVS stimulation.

A two–sample T–test was used to assess differences in the left thalamus functional connectivity between the two groups. First, we searched for any region showing a different connectivity in SF during the pre-CVS session as compared to the control group. Second, we checked whether the connectivity patterns of these regions returned to a normal range after CVS.

## Results

### Caloric Vestibular Stimulation Study

#### CVS–right ear

The CVS to the right ear affected neither the motor and verbal performances nor the pain and the somatosensory delusions (see S2 Fig in Supporting Information). The absence of nystagmus and vegetative sensation after the right ear irrigation was further investigated through a wide-ranging otolaryngologist examination. The clinical and instrumental check-up showed the presence of an acute sinusitis inflammation of the right sinuses and a complete obstruction of the internal and external canals of the ears. The lack of the nystagmus and vegetative sensations let us suppose that the obstruction of the internal and the external canals of the ear provoked by the mucus, caused a change in the somatosensory afferentation modifying the integrated sensory signal by sensory interaction. In turn it influenced the nystagmus and the self-motion perception evoked by CVS.

#### CVS–left ear

The modifications in motor skills, pain, and somatic delusions are showed in Fig 1 panel B. Five minutes after CVS, the patient reported a considerable reduction of pain and motor weakening. She also evaluated a consistent improvement in speech articulation. Interestingly, an additional improvement of all symptoms was observed 30 minutes after CVS with a remarkable reduction of the somatoparaphrenic delusions (Fig 1B).

The improvements in symptomatology were so impressive that SF commented: “I can’t believe, I can feel my hip again and my leg is not stuck to the neck anymore…I can also walk faster and the pain is much more tolerable…” (see also S1 Video and S2 Video in Supporting Information).

After one hour from CVS the improvements in all variables were stable. The beneficial effect of CVS begun to reduce after 3 hours and disappeared after 5 hours from stimulation. The very same pattern of effects was observed in the second left ear-stimulation applied 15 days later.

#### Independent scoring of the CVS effects on motor and speech functions

The Inter-Raters Reliability (IRR) was assessed by the Intra-Class Correlation (ICC) coefficient. This index is considered one of the most commonly-used statistics for assessing IRR for ordinal, interval, and ratio variables.[15]. Results showed very high IRR with an ICC = 0.79 in the PRE-CVS- VIDEO and a ICC = 0.75 in the POST-CVS-VIDEO.

The Anova with treatment (pre-post) as independent factor and SF’s performances on motor and verbal variables as depended factors, showed a significant effect of treatment (F(2,68) = 304, p < .001). Specifically the independent assessment evaluating the walk and the arm fluidity significantly improved after the CVS (F(2,68) = 150, p < .001, F(2,68) = 440, p < .001, respectively). Also verbal fluidity appeared improved (F(2,68) = 315, p < .001). See also S3 Fig in Supporting Information.

### fMRI study

#### Resting-state functional connectivity

**Fig 2:**
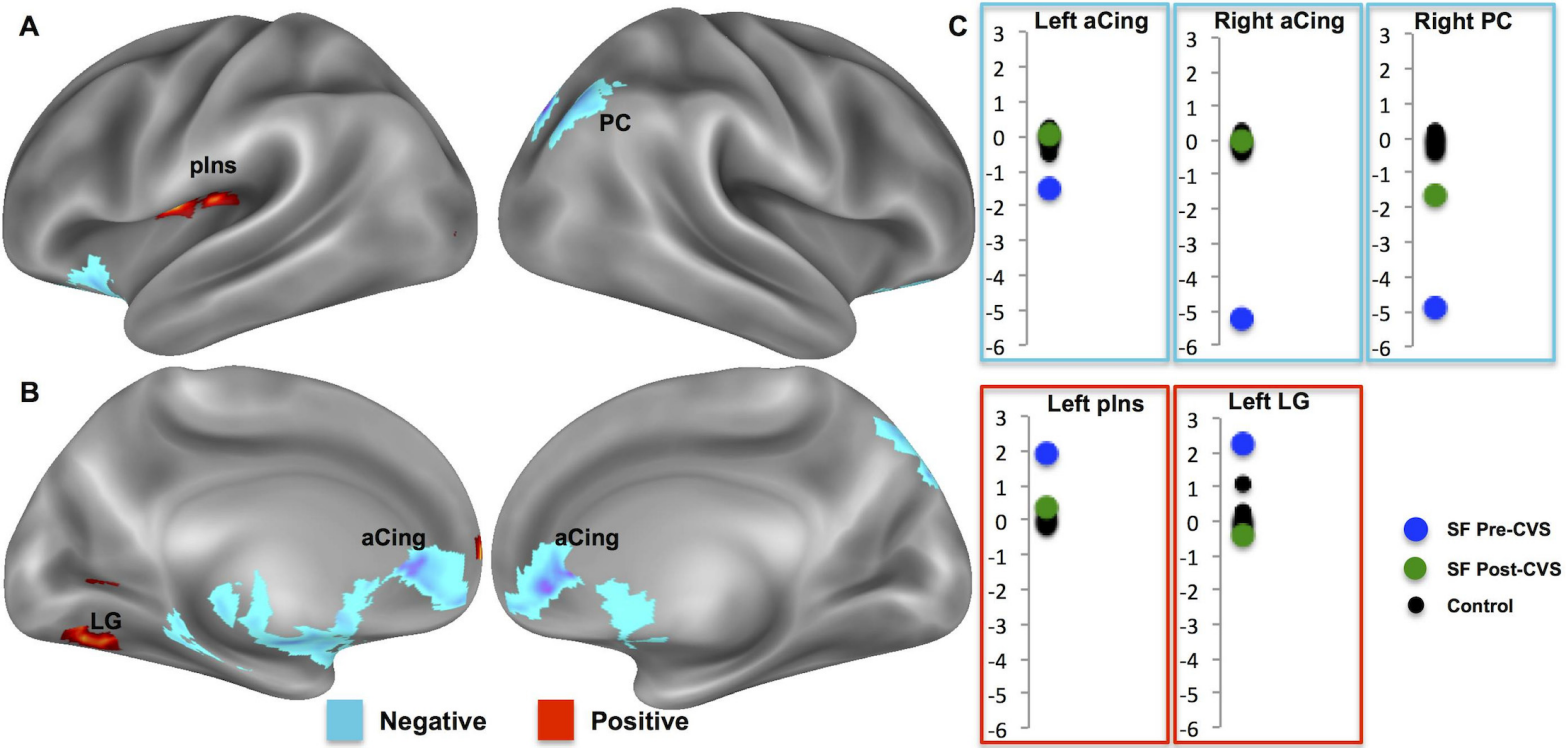
Functional connectivity of the left thalamus. (A–B) Comparison between the functional connectivity of the left thalamus between SF and a control group prior to CVS. Blue blobs = abnormally negative functional connectivity; Red blobs = abnormally positive connectivity. (C) Functional connectivity values (upper panel = negative and lower panel = positive) of the control group (black dots) and the patient (blue dot = Pre–CVS and green dot = Post–CVS). aCing = anterior cingulum; LG = lingual gyrus; PC = parietal cortex; pIns = posterior insula.

We used resting–state functional connectivity to assess the temporal correlations between the left thalamus and the rest of the brain in both SF and healthy subjects. Fig 2 shows the patient’s cortical regions exhibiting, during the CVS session, different connectivity patterns with the left thalamus as compared to the control group.

We found differences between the two groups, at a threshold of F(1,19) = 62.6, p = .01, FWE–corrected (extend threshold = 30 voxels), in a network of areas including the bilateral anterior cingulate cortex (aCing), the left posterior insula (pIns), the right parietal cortex (PC), as well as the left caudate and the left lingual gyrus (LG) (Fig 2). Some of these regions showed a positive functional connectivity at rest with the patient’s left thalamus (left pIns = 1.9, one–sample t–test vs. 0: T(1,19) = 21.97, p < .0001; left caudate = 2.4, one–sample t–test vs. 0: T(1,19) = 11.11, p < .0001; left LG = 2.2, one–sample t–test vs. 0: T(1,19) = 8.52, p < .0001, red blobs in Fig 2A and 2B). Other regions, such as the bilateral anterior cingulate cortex (left aCing = –1.5, one–sample t–test vs. 0: T(1,19) = – 8.37, p < .0001; right aCing = – 5.1, one–sample t–test vs. 0: T(1,19) = – 39.33, p < .0001) and the right parietal cortex (right PC = – 4.9, one–sample t–test vs. 0: T(1,19) = – 27.44, p < .0001) displayed negative functional connectivity with the left thalamus in the patient (blue blobs in Fig 2A and 2B). In the control group, no significant functional connectivity with the regions above was found.

During the post–CVS session, we looked at differences in the left thalamus connectivity between the two groups (patient vs. control) within the above-mentioned regions, i.e. those showing distinct connectivity patterns during the pre–CVS session. We found no significant differences in the left thalamus connectivity between SF and healthy participants. In other words, the patient’s pattern of connectivity within these regions, known to be involved in the cortical response to pain [16–18], returned to a normal range (see also S1 Table in Supporting Information). These results demonstrate that CVS stimulation produces a remarkable effect on regions of the brain associated with pain even after 45 minutes.

## Discussion

The patient described here was affected by chronic and stable CPSP after a stroke in the left hemisphere. SF suffered from an intolerable pain in the contralesional body part and impairments in the motor performance and speech articulation. Disorders of pain and motor functions have always been reported in CPSP patients. In addition with these clinical reports, patient SF reported severe somatosensory delusions of the right body part. This latter phenomenon is very rare; according to our knowledge there is only one case that described a patient who suffered from pain and somatosensory delusions following a left thalamic stroke [4].

As previously reported CPSP symptoms are often refractory to common treatments. An interesting attempt to reduce this kind of neuropathic pain has been successfully proposed by Ramachandran et al. (2007) who found that in two patients their pain was improved by cold vestibular caloric stimulation (CVS) [9]. These findings have been replicated in 4 out of 9 CPSP patients [10]. Furthermore Rode et al. (2012), described a fascinating case of a patient affected by left lateral medullary stroke [8]. Similarly to SF’s facial delusions, this patient reported a persistent somatosensory illusory sensation of enlargement, confined to the left side of his face (left macrosomatognosia) associated with a left facial anesthesia, and a neuropathic pain of the left trigeminal nerve. In this case the use of cold left CVS abolished the facial somatosensory illusion, for about 30 min, but had no effect on the left facial pain.

The patient that we have described here, was similar to those reported in the aforementioned literature on CPSP but, in addition, she experienced a rare pattern of spectacular somatosensory delusions in several parts of the body contralateral to the lesion. Our main aim was to test whether or not cold CVS could ameliorate both the pain and the somatic delusions.

In line with our expectations we found that 30 minutes after CVS, the symptomatology of SF was reduced supporting the beneficial use of CVS in the decrease of pain and confirming the data of McGeoch et al. (2008) and Ramachandran et al. (2007) [9–10]. As previously described by others [19] we found that left CVS improved the motor and speech articulation. Crucially we have also found a dramatic improvement of the somatosensory delusions of the right body parts. This latter finding seems to confirm the previous study of Rode et al. (2012) on the beneficial use of left cold CVS in the transitory remission of somatosensory illusory sensations.

In order to examine in depth the physiological mechanisms associated with the improved symptomatology of CPSP after CVS, we conducted an fMRI connectivity investigation. Results showed impressive changes in the left thalamus functional connectivity with the areas involved in the cortical response to pain.

Understanding the mechanism of CPSP is very complex and it involves both peripheral and central pathophysiological phenomenon. Since one century it has been accepted that central neuropathic pain arises as a direct consequence of lesions affecting the central nervous system and, accordingly to historical and more recent literature, it is widely recognized that lesions generating central pain typically involve the alteration of the spino-thalamo-cortical system [20–24]. Some authors have proposed a new taxonomy for specific pain syndromes commonly associated with central pain. For example Garcia-Larrea et al. (2010) suggested to use the term ‘parasylvian pain’ rather then “pseudothalamic syndrome” on the basis of the systematic occurrence of thermoalgesic sensory loss in patients with parasylvian or operculo-insular lesions [25]. The magnitude of thermal disturbances seems not a dissociated symptom in the central post-stroke pain of thalamic origin. It has also been reported that patients with central post-stroke pain of supratentorial origin including thalamic origin have greater deficits in sharpness and cold sensation, whereas patients with central post-stroke pain of infratentorial origin have greater deficits for warmth and hot pain [26]. Also, different patterns of symptoms and sensory deficits have been suggested in case of central post-stroke pain of non-thalamic origin, e.g. with spinal, brainstem or cortical lesions [5, 26]. The patient hereby described was not affected by any thermoalgesic sensory loss or tactile deficit and the medical case seems coherent with a genuine thalamic pain syndrome.

In spite of almost 100 years of investigation and a great amount of literature, the precise pathophysiological mechanisms leading to CPSP of thalamic origin are still debated. As skillfully reviewed by Sprenger et al. (2012), the use of modern brain mapping techniques endorsed a deeper investigation of the identification of the critical lesion locations responsible for central post-stroke pain [27]. Nevertheless, the low number of patients and the lack of control subjects did not permit an unambiguous explanation of the roles of the different thalamic nuclei [28,29].

It seems to us that many concepts explaining central post-stroke pain of thalamic origin, initially proposed by Head & Holmes, approach now the thermosensory disinhibition hypothesis [11–12].

In line with this model, we observed altered connectivity of the thalamus with the anterior cingulate cortex and the posterior insula. Interestingly, CVS was associated with improved patient symptoms and with a thalamic connectivity similar to the control group. It is worth noting that this change in connectivity was observed even after 60’ from the CVS.

An important step forward on the effect of CVS on thalamic patients has been made by Dieterich et al. (2005) in an impressive PET study [30]. Among several results, the authors found that the CVS of the ipsilesional left ear caused main activations within the left hemisphere located in the caudate nucleus, the lingual Gyrus and the anterior and medial parts of the insula. Of particular interest for our study is that Dieterich and co-workers found that the activation of the multisensory vestibular temporo-parietal cortex was significantly reduced in the left hemisphere when the ipsilesional ear was stimulated. It is noteworthy that in our connectivity study, in the PRE–CVS we have found that the aforementioned regions of the left hemisphere (namely the caudate, the lingual Gyrus and the insula) showed a positive functional connectivity with the left thalamus and that in the POST-CVS no significant functional connectivity with these regions was found.

Following this line of reasoning we can recap that in patients suffering from left thalamic lesions, the CVS reduce the activation of the multisensory vestibular temporo-parietal cortex and that in our patient this cortical activity’s reduction could bring to a normalization of the functional connectivity of the thalamus with other regions. According to the thermosensory disinhibition theory, central pain is a result of disruption of the normal inhibition of the pathways mediating thermal sensation in nociceptive systems. This theory suggests that central pain is an interoceptive thermoregulatory dysfunction due to alteration of the brain integration of pain and temperature.

We can speculate that, since the CVS reduces the activation of the cortex, the hyper activation became weaker and the inhibitory pathways can be temporary recovered. This suggestion can be further considered in light of the model offered by McGeoch et al. (2008) to explain the beneficial effect of CVS on CPSP [10]. The authors, proposed that the effect of CVS could arise due to two related (not mutually exclusive) mechanisms by which parieto-insular vestibular cortex (PIVC) might temporary rebalance the thermosensory information. The first mechanism suggests that PIVC might activate the adjacent thermosensory cortex in the dorsal posterior insula; the second proposes that PIVC is a part of a larger interoceptive system that exists to maintain homeostasis.

The disturbed body representation in somatoparaphrenia has been considered a result of disconnection between sensory inputs and cortical body areas [31]. However, we observed heightened activity in the somatosensory contralesional structures of our patient. CVS reduced this activity and was associated with a concomitant regression in behavioral delusions. CVS–induced improvement in motor skills also reflected trends observed for pain and somatosensory delusions; CVS appeared to “reactivate” the motor and somatosensory areas typically involved in finger tapping [32].

To understand the pathophysiology evoked by CVS, it is crucial to consider the timing of behavioral and neurological consequences. It took time for CVS to have a maximal effect on either response, differing from other forms of brain neurostimulation that have an on–off response (ie, optokinetic stimulation). In the case of CVS, symptom regression reached a maximum effect after 30–50 minutes. These data do not agree with a “reflex–like” model and suggest a change in neurotransmission as a consequence of CVS.

## Conclusions

In conclusion, we present data evidencing that cold CVS improved motor skills and reduced pain and somatosensory delusions in a CPSP female patient. The functional connectivity of the left thalamus with the anterior bilateral cingulate cortex and posterior left insula reverted from abnormal to within the range of our control group following stimulation.

Thus, this case study provides behavioral and imaging evidence on the benefit of using CVS for the treatment of pain and somatosensory delusions in chronic and severe CPSP. This study also provides insight into the mechanisms involved in CVS treatment. A prospective study evaluating the usefulness of this stimulation in the reduction of central pain symptomatology is warranted for targeted interventions for this challenging disorder.

## Supporting Information

S1 Fig MRI slices of patient SF. Subcortical and thalamic lesions as shown in multiple axial slices on the FLAIR sequence. LH: left hemisphere; RH: right hemisphere.
(DOCX)

S2 Fig Effect of CVS stimulation to the right ear. The effects of CVS in pain (1), motor function (2), somatoparaphrenia (3), and verbal articulation (4) In graphs 1-2-3, the Y axis shows the level of impairment scored with Visual Analogic Scale (VAS) (0 = no impairment; 100 = totally impaired). In graph 4 Y axis = 0 excellent (no altered articulation characteristics), 1 slight disorder, 2 moderate disorder, 3 severe disorder. In all panels post–CVS (5’) refers to measurements collected immediately after CVS and post–CVS (30’) refers to measurements collected 30 minutes after the CVS.
(DOCX)

S3 Fig Oneway Anova results of the independent assessment. In abscissa 0 = pre CVS and 1 = post CVS. In ordinate the Visual Analogic Scale scoring: 0–50 = bad performance; 60–100 = good performance.
(DOCX)

S1 Interview Structure interview of the monitoring sessions. (DOCX)

S1 Ref References of Table 1. (DOCX)

S1 Table Pre—and Post- treatment connectivity coefficients in SF and the functional connectivity values (mean and standard deviation) of the control group for each cortical region functionally connected with the left thalamus. (DOCX)

S1 Video (MOV)

S2 Video (MOV)
