# Supplementary material for: Caloric Vestibular Stimulation Reduces Pain and Somatoparaphrenia in a Severe Chronic Central Post-Stroke Pain Patient: A Case Study
Source: PLoS One. 2016 Mar 30;11(3):e0151213. doi: 10.1371/journal.pone.0151213 (PMC4814090; doi:10.1371/journal.pone.0151213)
Supplement: S1 Fig — Subcortical and thalamic lesions as shown in multiple axial slices on the FLAIR sequence. LH: left hemisphere; RH: right hemisphere. (DOCX) [file pone.0151213.s001.docx]

**S1 Fig. MRI slices of patient SF.**


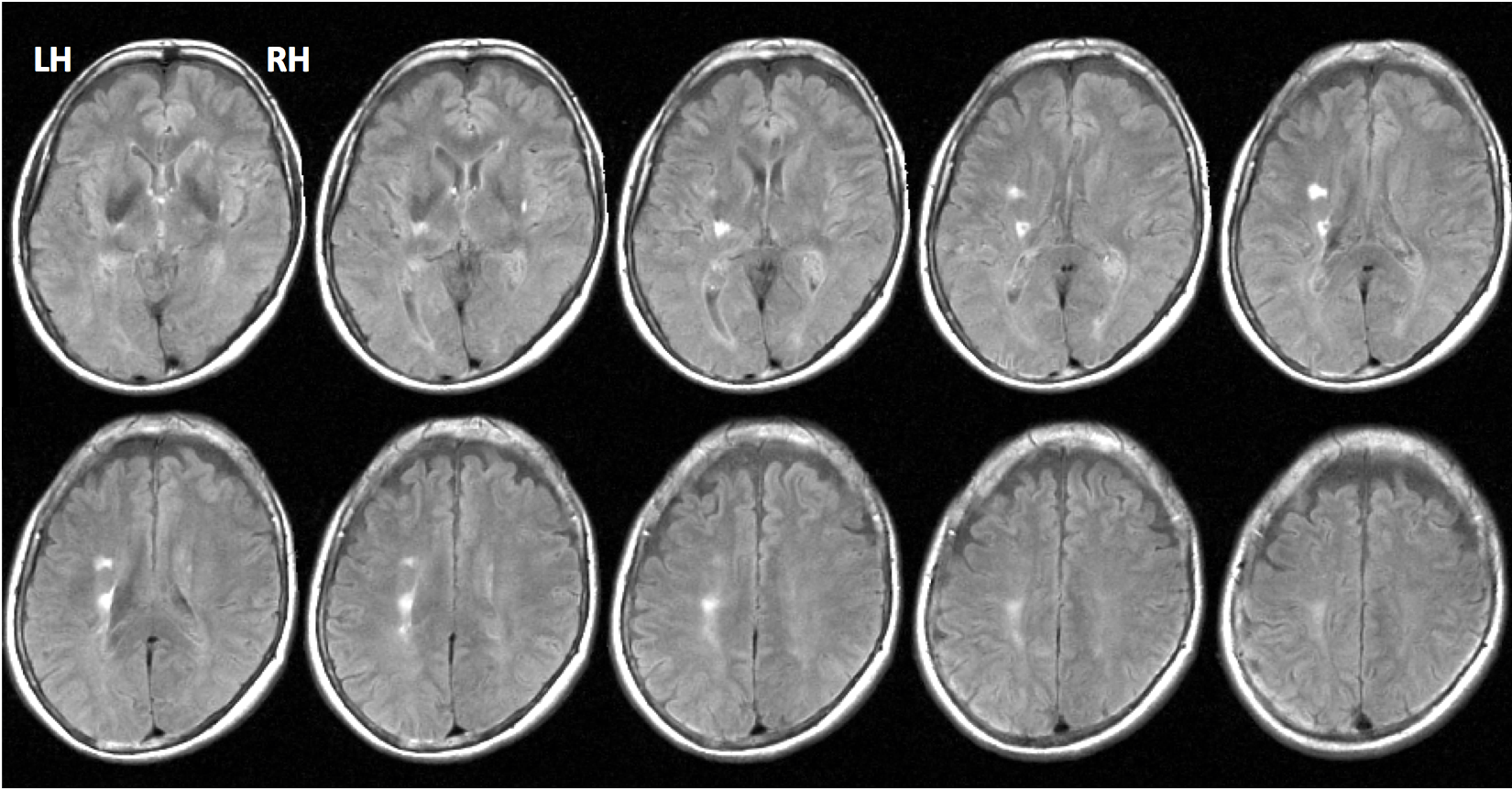


Subcortical and thalamic lesions as shown in multiple axial slices on the FLAIR sequence. LH: left hemisphere; RH: right hemisphere
