# Supplementary material for: Caloric Vestibular Stimulation Reduces Pain and Somatoparaphrenia in a Severe Chronic Central Post-Stroke Pain Patient: A Case Study
Source: PLoS One. 2016 Mar 30;11(3):e0151213. doi: 10.1371/journal.pone.0151213 (PMC4814090; doi:10.1371/journal.pone.0151213)
Supplement: S2 Fig — The effects of CVS in pain (1), motor function (2), somatoparaphrenia (3), and verbal articulation (4) In graphs 1-2-3, the Y axis shows the level of impairment scored with Visual Analogic Scale (VAS) (0 = no impairment; 100 = totally impaired). In graph 4 Y axis = 0 excellent (no altered articulation characteristics), 1 slight disorder, 2 moderate disorder, 3 severe disorder. In all panels post–CVS (5’) refers to measurements collected immediately after CVS and post–CVS (30’) refers to measurements collected 30 minutes after the CVS. (DOCX) [file pone.0151213.s002.docx]

**S2 Fig. Effect of CVS stimulation to the right ear.**

The effects of CVS in pain (1), motor function (2), somatoparaphrenia (3), and verbal articulation (4) In graphs 1-2-3, the Y axis shows the level of impairment scored with Visual Analogic Scale (VAS) (0 = no impairment; 100 = totally impaired). In graph 4 Y axis = 0 excellent (no altered articulation characteristics), 1 slight disorder, 2 moderate disorder, 3 severe disorder. In all panels post–CVS (5’) refers to measurements collected immediately after CVS and post–CVS (30’) refers to measurements collected 30 minutes after the CVS.
