# Supplementary material for: Caloric Vestibular Stimulation Reduces Pain and Somatoparaphrenia in a Severe Chronic Central Post-Stroke Pain Patient: A Case Study
Source: PLoS One. 2016 Mar 30;11(3):e0151213. doi: 10.1371/journal.pone.0151213 (PMC4814090; doi:10.1371/journal.pone.0151213)
Supplement: S3 Fig — In abscissa 0 = pre CVS and 1 = post CVS. In ordinate the Visual Analogic Scale scoring: 0–50 = bad performance; 60–100 = good performance. (DOCX) [file pone.0151213.s003.docx]

**S3 Fig. Oneway Anova results of the independent assessment.**

In abscissa 0 = pre CVS and 1 = post CVS. In ordinate the Visual Analogic Scale scoring: 0-50 = bad performance; 60-100 = good performance
