# Supplementary material for: Caloric Vestibular Stimulation Reduces Pain and Somatoparaphrenia in a Severe Chronic Central Post-Stroke Pain Patient: A Case Study
Source: PLoS One. 2016 Mar 30;11(3):e0151213. doi: 10.1371/journal.pone.0151213 (PMC4814090; doi:10.1371/journal.pone.0151213)
Supplement: S1 Interview — (DOCX) [file pone.0151213.s004.docx]

**S1 Monitoring Interview**

1) Common health questions

- Q1 How do you feel now
- Q2 Can you answer to some question regarding your symptoms?

2) Questions on Pain

- Q3 From 0 to 10, how do you feel the pain in your right:

Face

Mouth

Hip

Shoulder

Arm

Leg

3) Questions on Motor Skill

- Q4 From 0 to 10, how well can you move your mouth
- Q5 From 0 to 10, how well can you move your right:

Hip

Shoulder

Arm

Leg

Fingers

4) Questions on Delusions

- Q6 From 0 to 10, how much do you feel that your:

face is enlarged

teeth are moving

teeth are in the foot

hip is stuck to the arm

leg is elongated
