# Supplementary material for: Caloric Vestibular Stimulation Reduces Pain and Somatoparaphrenia in a Severe Chronic Central Post-Stroke Pain Patient: A Case Study
Source: PLoS One. 2016 Mar 30;11(3):e0151213. doi: 10.1371/journal.pone.0151213 (PMC4814090; doi:10.1371/journal.pone.0151213)
Supplement: S1 Ref — (DOCX) [file pone.0151213.s005.docx]

**S1 Ref.**

**References of Table 1**

**Orientation**

Space - Time

Benton AL. Sivan AB. (1994). *Contributions to Neuropsychological Assessment*: A Clinical Manual 2^nd^ Edition. New York, USA. Oxford University press.

**Attention**

Visual Search

Della Sala S, Laiacona M, Spinnler H, Ubezio C (1992) *A cancellation test : its reliability in assessing attentional deficits in Alzheimer's disease.* Psychological Med. 22:885-901.

Go-no go test -.

Barbarotto R, Laiacona M, Frosio R, Vecchio M, Farinato A, Capitani E (1998) *A normative study on visual reaction times and two Stroop colour-word tests*. Ital J Neurol Sci. Jun;19:161-70.

**Language**

Token Test

De Renzi E, Faglioni P. (1978) *Normative data and screening power of a shortened version of the Token Test.* Cortex. 14:41-9.

Fluency (semantic)- Fluency (lexical) Avanzi S, Posteraro L, Gugliotta M, Lombardi F, Cavatorta S., Mazzucchi A (1997) *Aspetti quantitativi e qualitativi della fluenza verbale I. Nei soggetti normali.* Archivio di Psicologia, Neurologia e Psichiatria, 48: 85-108.

**Apraxia**

Ideomotor -Ideational

De Renzi, Pieczuro A, Vignolo, L. (1966) *Oral Apraxia and Aphesia*. Cortex 2; 50-73.

Constructional

Spinnler H and Tognoni (1987) *Standardizzazione e taratura italiana di test neuropsicologici*. Ital J Neurol Sci 8:1–120.

**Agnosia**

Categorical- Functional

De Renzi E, Lucchelli F. (1993) *The fuzzy boundaries of apperceptive agnosia*. Cortex 29 :187-215.

**Memory**

Corsi Span and Verbal Span

Orsini A, Grossi D, Capitani E, Laiacona M, Papagno C, Vallar G (1987) *Verbal and spatial immediate memory span: normative data from 1355 adults and 1112 children*. Ital J Neurol Sci. 8:539-48.

**Executive Functions**

BADS

Antonucci G, Spitoni G, Orsini A, d'Olimpio F, e Cantagallo A(2014) *Behavioural Assessment of the Dysexecutive Syndrome* *Italian Valitade form*. O.S. Organizzazioni Speciali, Firenze.

Tower Of London

Allamanno N, Della Sala S, Laiacona M, Pasetti C., Spinnler, H. (1987) *Problem-solving ability in aging: normative data on a nonverbal test* Ital. J. Neurol. Sci. 8, 111-119.

WAIS-R

Wechsler, D. (1981), *WAIS-R. Scala d’Intelligenza Wechsler per Adulti-Riveduta*. Tr. it. O.S. Organizzazioni Speciali, Firenze.
