# Supplementary material for: Caloric Vestibular Stimulation Reduces Pain and Somatoparaphrenia in a Severe Chronic Central Post-Stroke Pain Patient: A Case Study
Source: PLoS One. 2016 Mar 30;11(3):e0151213. doi: 10.1371/journal.pone.0151213 (PMC4814090; doi:10.1371/journal.pone.0151213)
Supplement: S1 Table — (DOCX) [file pone.0151213.s006.docx]

**S1 Table.** Pre - and Post- treatment connectivity coefficients in SF and the functional connectivity values (mean and standard deviation) of the control group for each cortical region functionally connected with the left thalamus

|  | | SF | | Control | |
| --- | --- | --- | --- | --- | --- |
|  |  | Pre-CVS | Post-CVS | mean | s.d. |
| Left | pIns | 1.94 | 0.36 | 0.05 | 0.09 |
| Left | aCing | -1.50 | 0.06 | -0.01 | 0.18 |
| Right | aCing | -5.22 | -0.03 | -0.05 | 0.13 |
| Left | LG | 2.22 | -0.39 | 0.08 | 0.26 |
| Right | PC | -4.90 | -1.62 | -0.08 | 0.18 |
